# Do no harm: can school mental health interventions cause iatrogenic harm?

**DOI:** 10.1192/bjb.2023.9

**Published:** 2023-10

**Authors:** Lucy Foulkes, Argyris Stringaris

**Affiliations:** 1 University of Oxford, UK; 2 UCL, UK

**Keywords:** Mental health, adolescence, school interventions, iatrogenic harm, adverse effects

## Abstract

In recent years, there have been extensive efforts in secondary schools to prevent, treat and raise awareness of adolescent mental health problems. For some adolescents, these efforts are essential and will lead to a reduction in clinical symptoms. However, it is also vital to assess whether, for others, the current approach might be causing iatrogenic harm. A growing body of quantitative research indicates that some aspects of school-based mental health interventions increase distress or clinical symptoms, relative to control activities, and qualitative work indicates that this may be partly due to the interventions themselves.

There is a widely held belief among academics, clinicians and policy makers that secondary schools should help to prevent and treat adolescent mental health problems. This perspective has been bolstered by an ongoing government initiative to train senior mental health leads, promote whole-school approaches to mental health and increase access to low-intensity psychological interventions in all schools. This ‘therapeutic turn’ in education makes intuitive sense, for a number of reasons. Adolescents spend many of their waking hours in school. School-based approaches increase access for those who might not otherwise seek help and mean adolescents do not need to travel to a clinical setting for treatment, which can be time-consuming or stigmatising. It is also what adolescents themselves want: a 2021 survey found that 93% of 11–19-year-olds thought the topic of mental health should be taught at school.^1^ Last, trial evidence suggests that school-based interventions can be effective at reducing mental health problems.^2^ Effect sizes are generally small but could still equate to meaningful, impactful change if scaled up across hundreds or thousands of schools.^3^ Given that rates of adolescent mental health problems have increased in the past decade and that such problems often start during this age period, it makes good sense that secondary schools are viewed as ideal sites of prevention, treatment and support.

## The problem with school mental health interventions

Unfortunately, this vision has not yet been translated into a reality, and we argue here that the potential benefits of school mental health interventions can also be their weaknesses. This is particularly relevant for universal interventions and approaches, in which all students are exposed to the same content (for example, whole-class lessons or school-wide awareness-raising initiatives). We argue that the generalised and widespread nature of these efforts means that some students could be taught information or strategies that are not only unhelpful or irrelevant to them but that may actively cause harm. Indeed, this concern is still relevant for some targeted small-group or one-to-one interventions. Below, we lay out emerging evidence that school-based approaches can cause harm in at least some adolescents and consider the mechanisms by which this might happen.

A growing body of quantitative research indicates that some school-based mental health interventions can cause iatrogenic harm (adverse effects from the treatment approach itself). Psychological interventions more generally can lead to a range of harms,^4^ but this research in schools specifically demonstrates an increase in internalising symptoms relative to control groups. A meta-analysis of anti-bullying interventions found that, in some studies, students who were taught cognitive–behavioural therapy (CBT) skills experienced an increase in internalising symptoms relative to control groups.^5^ A randomised control trial of another CBT-based school intervention also found an increase in internalising symptoms in the intervention group compared with those who had their usual lessons.^6^ These findings tell us there were instances when, on average, a participant was worse off receiving the intervention than not receiving it – i.e. this is evidence of iatrogenic harm.

It is also important to consider whether there are subgroups of adolescents who will experience harms from interventions, which may be masked when findings are averaged. For example, a recent trial assessing mindfulness lessons in secondary schools found that overall there was no change in depressive symptoms in the intervention (or control) group, but that adolescents with elevated levels of mental health symptoms at baseline experienced a small increase in depressive symptoms after the intervention, relative to those who had their usual social-emotional teaching.^7^ This should indicate to all researchers and clinicians that even if there is evidence that a school-based intervention is effective or ineffective on average, there may still be a minority of participants to whom it can actively cause harm. Powering trials sufficiently to allow for testing of such subgroup effects is as important as it is challenging.

These findings should not be surprising. It has long been recognised that the psychological therapies on which interventions are based can cause harm in a minority of individuals, including adolescents.^8,9^ There is also an established body of literature demonstrating harms from public health interventions.^4^ As school-based mental health interventions similarly aim to change adolescents’ thoughts, feelings or behaviours, it is reasonable that this too might have negative effects for some individuals.

## Possible mechanisms

To date, there has been very little investigation into why harms such as symptom increase occur in school-based mental health interventions. Here, we speculate that one relevant mechanism might be that interventions inadvertently encourage adolescents to ruminate on their negative thoughts and emotions. Indeed, qualitative studies highlight that although some adolescents find school mental health interventions helpful, others say the focus on negative thoughts made them feel more stressed and unhappy.^10^ Relatedly, if an adolescent is encouraged to label their negative thoughts and emotions with psychological or psychiatric terminology in school interventions, this might lead to changes in self-concept (e.g. ‘I have anxiety’) and changes in behaviour (e.g. avoidance) that ultimately increase distress and other symptoms in some adolescents.^11^

The unique developmental features of adolescence may also be relevant. Adolescents are especially susceptible to peer influence, and school-based mental health interventions commonly occur in groups. It is well established that adolescents can influence each other's negative moods and can learn problematic behaviour from each other (sometimes known as ‘deviancy training’). It is therefore a reasonable hypothesis that encouraging adolescents to discuss negative thoughts, feelings and behaviours in group settings, as is so common in school-based interventions, could lead to an increase in these experiences in others via peer influence and social learning.

## Public health concern

The risk of iatrogenic harm and adverse effects from school-based mental interventions, even in a minority of adolescents, amounts to a potentially vast public health problem. There are over 3.5 m secondary school pupils in England. In clinical settings, approximately 3–10% of patients experience symptom deterioration during therapy (this can be due to number of factors, including iatrogenic harm but also the natural course of the disorder in those for whom the treatment is ineffective).^8^ As we have shown in a recent simulation-based study,^3^ even if the number who experience symptom deterioration as a result of school-based mental health interventions is relatively small, if these approaches are scaled up nationally – as is being encouraged – this could affect hundreds of thousands of adolescents. In other words, just as statistically small positive effects can lead to large benefits for society as a whole, statistically small negative effects can lead to considerable harms at scale.

Even if school-based interventions are only ineffective, as is often the case with universal approaches in particular,^12^ this is still a serious concern, as it amounts to an opportunity cost (i.e. foregone benefits of options not chosen). Time is taken away from other activities that could potentially be more enjoyable or more conducive to better mental health for adolescents, such as physical exercise, extra time to sleep in the morning or free time to socialise. We should be very cautious about the idea that providing any mental health intervention in a school is always better than not providing one at all.

## Conclusions

There is currently a pervasive assumption that school-based mental health interventions are beneficial for all adolescents. The possibility that some individuals may deteriorate or experience harm as a result of such efforts has been almost entirely neglected. As a matter of urgency, research should begin that explores and documents what intervention harms might look like in school settings and which adolescents are most at risk. In time, all studies assessing school-based mental health interventions should measure and report cases of symptom deterioration and other adverse effects as standard, as happens with clinical trials.^9^ More importantly, it should become standard to have a plan of what to do with adolescents who deteriorate during these interventions – for example, to conduct follow-up assessments and offer group or individual interventions as necessary. When there is an evidence base demonstrating which individuals are more likely to experience harm from school interventions, then more tailored, effective support can be offered. Future research should also explore the mechanisms by which iatrogenic harm and adverse effects might happen in school settings. Together, such studies will allow the field to develop school-based mental health interventions that are the most beneficial, and least harmful, for all adolescents.

## About the authors

**Lucy Foulkes** is a Prudence Trust research fellow in the Department of Experimental Psychology, University of Oxford, UK. She is also an honorary lecturer in the Department of Clinical, Educational and Health Psychology, UCL, UK and a senior research fellow at the Anna Freud National Centre for Children and Families, UK. **Argyris Stringaris** is a Child and Adolescent Psychiatry in the Divisions of Psychiatry and Psychology and Language Sciences, UCL, UK, and at the 1st Department of Psychiatry, National and Kapodistrian University of Athens, Athens, Greece.
