# Do no harm: Can school mental health interventions cause iatrogenic harm? – ERRATUM

**DOI:** 10.1192/bjb.2023.22

**Published:** 2023-10

**Authors:** Lucy Foulkes, Argyris Stringaris

**Keywords:** Mental health, adolescence, school interventions, iatrogenic harm, adverse effects, erratum

This article was originally published with a spelling error in the sentence “We should be very cautious about the idea that providing any mental health intervention in a school is always better than not providing one at all.” This has now been corrected and this erratum published. We apologise for the error.
